# Prognostic significance of the pretreatment pan-immune-inflammation value in cancer patients: an updated meta-analysis of 30 studies

**DOI:** 10.3389/fnut.2023.1259929

**Published:** 2023-10-02

**Authors:** Yu Hai-Jing, Ren Shan, Xia Jie-Qiong

**Affiliations:** Department of International Nursing School, Hainan Medical University, Haikou, Hainan, China

**Keywords:** cancer, pan-immune-inflammation value, overall survival, progression-free survival, meta-analysis

## Abstract

**Background:**

The pan-immune-inflammation value (PIV) has been reported as a promising prognostic biomarker in multiple cancers but still remains inconclusive. The objective of this study is to systematically investigate the association of the pretreatment PIV with survival outcomes in cancer patients, based on available literature.

**Methods:**

Online databases including PubMed, Embase and the Web of Science were thoroughly searched for studies evaluating the prognostic role of the pretreatment PIV in cancers from the inception to June 2023. Hazard ratios (HRs) with 95% confidence intervals (CIs) were always assessed using a random-effects model. Statistical analyses were performed using Stata 12.0.

**Results:**

Thirty studies were finally included after comprehensively study searching. In total, 8,799 cancer patients were enrolled in this meta-analysis. The pooled results demonstrated that patients in the high PIV group had a significantly poorer overall survival (HR = 2.07; 95%CI: 1.77–2.41; *I*^2^ = 73.0%) and progression-free survival (HR = 1.83; 95%CI: 1.37–2.45; *I*^2^ = 98.2%) than patients in the low PIV group. The prognostic significance of the PIV score on overall survival and progression-free survival was observed across various geographical regions, tumor stages and treatment strategies. Sensitivity analyses supported the stability of the above combined results.

**Conclusion:**

This meta-analysis demonstrated that the pretreatment PIV could be a non-invasive and efficacious prognostic biomarker for cancer patients.

## 1. Introduction

With the global population and the proportion of elderly people growing, cancer has become one of the leading causes of death worldwide (1). Although the development of surgery and medical treatment has made great progress in cancer patients, the prognosis for these patients remains not yet satisfactory (2). Therefore, based on the estimated survival time of cancer patients, it is essential to develop individualized and effective treatment strategies to improve their chances of survival. Currently, anti-tumor therapy relies primarily on a conventional staging system. Nevertheless, in clinical practice, the staging system alone is not able to support treatment decision-making as well as prognosis assessment well (3, 4). It is therefore urgent to construct novel prognostic markers to guide more precise treatment for cancer patients.

The accumulating evidence suggests that host inflammation and immune status play an important role in the progression, treatment response and survival outcomes of cancer patients (5, 6). Based on this insight, several inflammation/immune-related biomarkers have been developed to predict the clinical outcomes of cancer patients, such as neutrophil to lymphocyte ratio (NLR) (7), platelet to lymphocyte ratio (PLR) (8) and monocyte to lymphocyte ratio (MLR) (9). Recently, a newly developed prognostic biomarker- the pan-immune-inflammation value (PIV), has garnered significant interest of clinicians (10). PIV integrates neutrophils, platelets, monocytes and lymphocyte together, and has been reported to be a better prognostic predictor than these simple biomarkers, including NLR, PLR and MLR (11, 12). To be specific, PIV is calculated using serum neutrophil, platelet, monocyte and lymphocyte (neutrophil x platelet x monocyte/ lymphocyte), which was first introduced by Fuca et al. (13) in 2020 as a prognostic index for metastatic colorectal cancer receiving chemotherapy combined with target therapy. After that, the prognostic role of the PIV has been explored in various cancers (14–16). A recent meta-analysis of 15 studies demonstrated that a high PIV was associated with a poor prognosis in cancer patients (17). Nonetheless, it is important to note that some common tumor types, such as pancreatic cancer and hepatic cancer, were not available in this meta-analysis. Besides, abstract without sufficient data was also included for analysis. These factors undoubtedly have a certain impact on the universality and reliability of the results.

As growing body of additional research has been addressed to further explore the prognostic value of PIV in cancer patients. We therefore performed an updated pooled analysis to systematically explore the relationship between the pretreatment PIV and survival outcomes in cancer patients.

## 2. Methods

### 2.1. Search strategy

This meta-analysis was conducted as per the PRISMA guidelines (18) (see PRISMA checklist in the Supplementary Information) to identify literature evaluating the association of pretreatment PIV with survival outcomes in cancer patients. Related studies from the Web of Science, PubMed, and Embase were thoroughly examined from the inception to June 30, 2023. The key word “pan-immune-inflammation value” was applied to search potential studies. During the search process, studies published in any language were included. In addition, references to enrolled studies and related reviews were prudently scanned for additional reporting. The search was performed by two investigators (Y-HJ and RS) independently.

### 2.2. Study selection

The inclusion criteria were as follows: (1) patients were pathologically diagnosed as cancer; (2) patients were divided into two groups according to the pretreatment PIV cut-off value; (3) studies investigated the relationship between the pretreatment PIV and survival outcomes of cancer patients. The exclusion criteria were: (1) letters, case reports, abstracts or reviews; (2) duplicated studies.

### 2.3. Data extraction and quality assessment

Data extraction and subsequent cross-checks were performed by two independent reviewers (YH-J and RS). Information extracted from included studies was as follows: first author, year of publication, country, study interval, sample size, cancer type, selection method, cut-off value, period of blood collection, information on exclusion of diseases affecting blood parameters, age, sex, tumor stage, treatment strategy, survival data and follow-up time. The quality assessment of included literature was evaluated via the method by Lin et al. (19). After careful evaluation from 9 domains, a study could get a total score ranging from 0 to 9. Quality assessment was not used as exclusion criterion for included studies.

### 2.4. Outcome assessment

In this study, the primary endpoint was to explore the relationship between the pretreatment PIV and survival outcomes in cancer patients. Long-term survival outcomes included overall survival (OS), progression-free survival (PFS), disease-free survival (DFS) and recurrence-free survival (RFS). Since DFS, RFS and PFS share the similar endpoints, they were analyzed together as one outcome, PFS, as previously suggested (20, 21).

### 2.5. Statistical analysis

Stata 12.0 statistical software was used to perform all the statistical analyses. Hazard ratios (HRs) with 95% confidence intervals (CIs) reported from multivariate analyses were preferentially used to incorporate survival outcomes. Otherwise, univariate assessments were the sources of effect sizes. In addition, for studies whose survival data were not directly available, corresponding HRs with 95% CIs were extracted from the survival curves through the methods reported by Tierney et al. (22). In the present study, I^2^ statistics were utilized to evaluate inter-study heterogeneity, and a random-effects model was always performed, which accounts for variance across included studies (23, 24). Subgroup analyses and meta-regression analyses were applied to explore the sources of heterogeneity. Leave-one-out sensitivity analyses were utilized to assess the reliability of pooled results. Possible publication bias was evaluated using Begg’s test. If there was a significant publication bias, a trim and fill analysis was employed to assess the impact of it on the pooled result. *p* values <0.05 were considered statistically significant.

## 3. Results

### 3.1. Study characteristics

**Figure 1:**
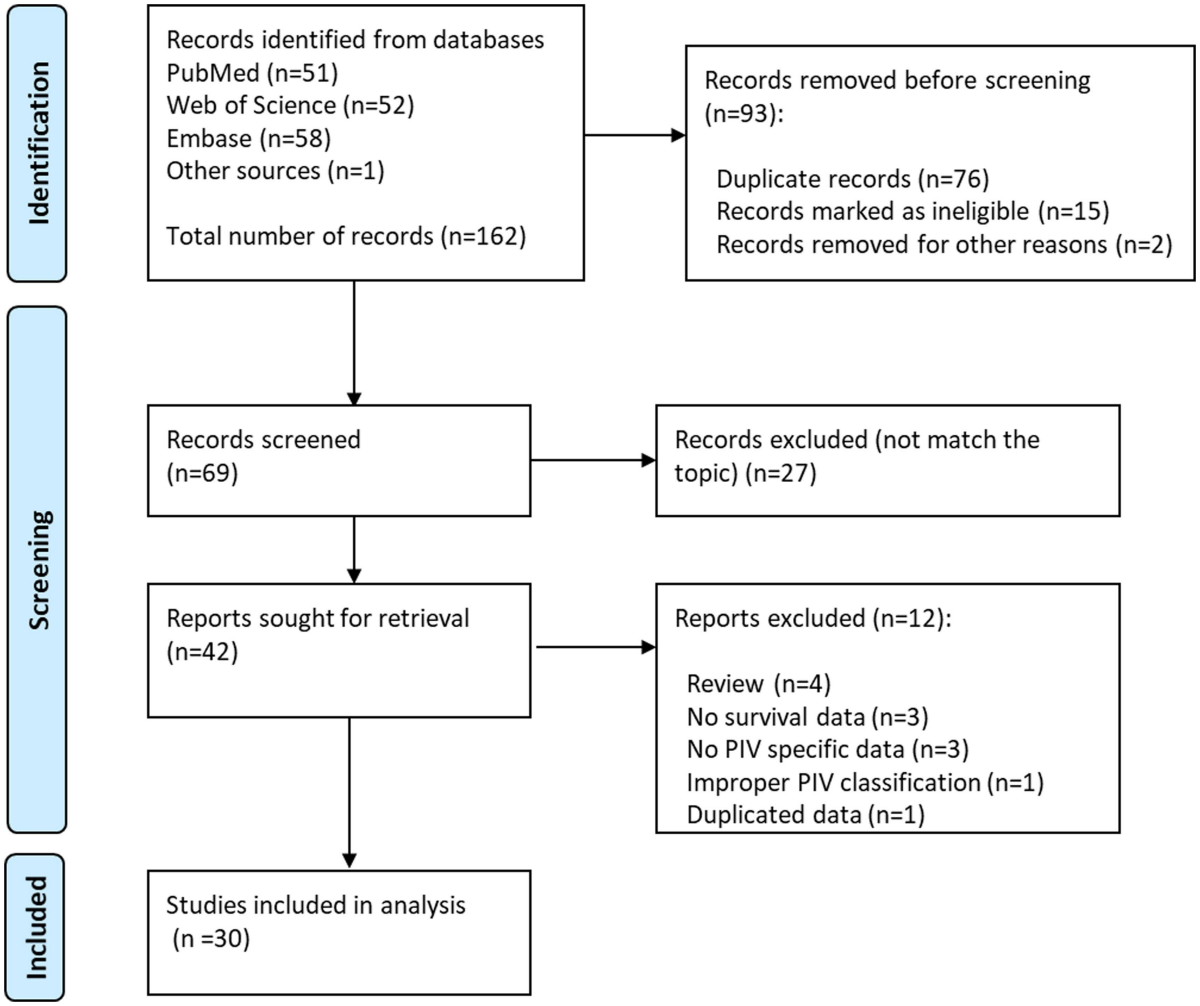
The PRISMA flowchart of study selection.

The initial search of online databases yielded a total of 162 records. By removing duplicated studies, and reviewing titles, abstracts and full-text studies, 30 studies (11–16, 25–48) with 32 cohorts were ultimately incorporated in our meta-analysis (Figure 1), The main characteristics of these studies were shown in Tables 1, 2. In total, 8,799 participants from China, Germany, Italy, Japan, Slovenia, Spain and Turkey were enrolled in the present study. These studies were published from 2020 to 2023, with a sample size ranging from 49 to 1,312. The most common cancer type was gastrointestinal cancer, followed by breast cancer and lung cancer. As regards blood parameters, the period of blood collection before treatment ranged from 1 day to 1 month, and most of the included studies did not mention the exclusion of diseases affecting hematological parameters. The cut-off value of PIV ranged from 164.6 to 600.0. In terms of main primary treatments, surgery was performed in 8 cohorts, chemo/radiotherapy was performed in 8 cohorts and immunotherapy contained treatment was performed in 7 cohorts. The median follow-up time ranged from 9.5 to 78.4 months. The literature quality of these studies was good with a median score of 8 (range: 7–9, Supplementary Table S1).

**Table 1:** Basic information of included studies.

References	Country	Study design	Study interval	Cancer type	Sample size	Age, years (Median/ Mean)	Sex (Male/Female)	Selection method	Cut-off value	The period of blood collection	Exclusion of diseases affecting blood parameters
Baba et al. (14) (training)	Japan	S;R	2005–2020	Esophageal cancer	433	66.5 ± 8.5	376/57	ROC	164.6	Within 1 week before treatment	NA
Baba et al. (14) (validation)	Japan	S; R	2005–2020	Esophageal cancer	433	66.3 ± 8.9	384/49	ROC	164.6	Within 1 week before treatment	NA
Chen et al. (15)	China	S; R	2014–2019	Lung cancer	94	48 (Range, 18–76)	55/39	Median	364	Within 3 weeks before treatment	Yes
Corti et al. (16)	Italy	M; R	2014–2020	Colorectal cancer	163	NA	90/73	MSR	492	Within 1 week before treatment	NA
Demir et al. (25)	Turkey	S; R	2006–2020	Breast cancer	243	36 (Range, 21–40)	0/243	Median	301	Before treatment	Yes
Efil et al. (26)	Turkey	S; R	2008–2016	Colorectal cancer	304	62 (Range, 19–91)	182/122	Median	491	Within 2 weeks before treatment	NA
Fucà et al. (13)	Italy	M; R	2008–2018	Colorectal cancer	438	62 (IQR, 53–68)	275/163	MSR	380	Before treatment	NA
Fucà et al. (27)	Italy	S; R	2010–2020	Melanoma	228	NA	142/86	MSR	600	Before treatment	NA
Gambichler et al. (28)	Germany	S; R	NA	Merkel cell carcinoma	49	77 (Range, 51–95)	25/24	ROC	372	Within 1 week at diagnosis	NA
Guven et al. (29)	Turkey	S; R	2016–2020	Multiple cancers	120	61 (IQR, 54–67)	86/34	Median	513.4	Before treatment	NA
Guven et al. (30)	Turkey	S; R	2005–2020	Head and neck cell carcinoma	199	59 (IQR, 53–67)	180/19	ROC	404	Within 1 week before treatment	NA
Karadağ et al. (11)	Turkey	S; R	2013–2021	Hepatocellular carcinoma	120	64 (IQR, 55–72)	101/19	Median	286.15	Before treatment	Yes
Kucuk et al. (31)	Turkey	M; R	2010–2021	Lung cancer	89	61 (Range, 37–79)	75/14	ROC	417	Within 1 week before treatment	Yes
Liang et al. (32)	China	S; R	2013–2016	Colorectal cancer	753	NA	473/280	ROC	231	Within 1 week before treatment	NA
Ligorio et al. (33)	Italy	S; R	2014–2020	Breast cancer	57	53 (Range, 26–78)	0/57	Median	285	Before treatment	Yes
Lin et al. (19)	China	S; R	2010–2012	Breast cancer	1,312	48 (IQR, 41–57)	0/1312	MSR	310.2	Within 1 week before treatment	Yes
Mesti et al. (34)	Slovenia	S; R	2018–2020	Melanoma	129	66.2 (Range, 30.1–84.5)	84/53	Median	390	Before treatment	Yes
Pérez-Martelo et al. (35)	Spain	S; R	2015–2018	Colorectal cancer	130	68.8 (Range, 26–88)	96/34	MSR	380	Within 1 month before treatment	NA
Provenzano et al. (36)	Italy	S; R	2008–2020	Breast cancer	78	NA	0/78	Median	228	Within 1 week before treatment	NA
Qi et al. (37)	China	S;P	2019–2022	Esophageal Cancer	51	62 (Range, 39–75)	44/7	ROC	232.8	Before treatment	NA
Sahin et al. (38)	Turkey	S; R	2008–2019	Breast cancer	743	48.0 (Range, 22.0–83.5)	0/743	ROC	306.4	Within 2 weeks before treatment	Yes
Sato et al. (39)	Japan	S; R	2013–2020	Colorectal cancer	86	70 (Range, 37–93)	50/36	ROC	209	Before treatment	Yes
Sato et al. (40)	Japan	S; R	2000–2019	Colorectal cancer	758	NA	466/292	ROC	376	Before treatment	Yes
Susok et al. (41)	Germany	S; R	NA	Melanoma	62	67 (Range, 18–85)	40/22	ROC	455	Before treatment	NA
Topkan et al. (42)	Turkey	S; R	2007–2020	Glioblastoma Multiform	204	58 (Range, 21–80)	135/69	ROC	385	The first day of treatment	Yes
Topkan et al. (43)	Turkey	S; R	2007–2020	Pancreatic adenocarcinoma	178	57 (Range, 26–79)	137/41	ROC	464	The first day of treatment	Yes
Wang et al. (44)	China	S; R	2010–2018	Gastric cancer	89	59 (Range, 32–78)	69/20	ROC	218.7	Before treatment	NA
Yazgan et al. (45)	Turkey	S; R	2010–2021	Prostate cancer	114	64 (IQR, 60–70)	114/0	Median	366	Within 1 month before treatment	NA
Yeh et al. (46)	China	S; R	2005–2017	Oral cavity cell carcinoma	853	53.5	780/73	ROC	268	Before treatment	NA
Yekedüz et al. (47)	Turkey	M; R	NA	Renal cell carcinoma	152	60 (IQR, 54–67)	117/35	MSR	372	Within 1 week before treatment	NA
Zeng et al. (48) (training)	China	M; R	2018–2020	Lung cancer	53	NA	34/19	Median	581.95	Before treatment	NA
Zeng et al. (48) (validation)	China	M; R	2015–2021	Lung cancer	84	NA	75/9	Median	581.95	Before treatment	NA

Retro, retrospective study; Pro, prospective study; M, multiple center; S, single center; ROC, receiver operator characteristic curve; MSR, maximally selected rank; IQR, interquartile range; NA, not available.

**Table 2:** Survival information of included studies.

References	Sample	Treatment strategy	Tumor stage	Survival outcomes	Multivariate analysis	Median follow-up time, months
Baba et al. (14) (training)	433 (225:208)	Surgery	Mixed	OS	No	NA
Baba et al. (14) (validation)	433 (210:223)	Surgery	Mixed	OS	Yes	58.8
Chen et al. (15)	94 (47:47)	First-line ALK inhibitor	Mixed	OS;PFS	Yes; Yes	47.0 (IQR, 38.5–55.5)
Corti et al. (16)	163 (63:100)	Immunotherapy	Metastatic	OS;PFS	Yes; Yes	31
Demir et al. (25)	243 (122:121)	Surgery	Mixed	OS	No	NA
Efil et al. (26)	304 (152:152)	Surgery	Non-metastatic	OS; DFS	Yes; Yes	NA
Fucà et al. (13)	438 (230:208)	Chemotherapy combined with target therapy	Metastatic	OS; PFS	Yes; Yes	38.4 (IQR, 27.4–50.9)
Fucà et al. (27)	228 (51:177)	Immunotherapy combined with target therapy	Metastatic	OS; PFS	Yes; Yes	35.3
Gambichler et al. (28)	49 (31:18)	Mixed therapy	Non-metastatic	RFS	No	NA
Guven et al. (29)	120 (60:60)	Immunotherapy	Metastatic	OS; PFS	Yes; Yes	9.62
Guven et al. (30)	199 (101:98)	Chemoradiotherapy	Non-metastatic	OS;DFS	Yes; Yes	71.59
Karadağ et al. (11)	120 (60:60)	Mixed therapy	Mixed	OS	Yes	9.5 (IQR:3–23)
Kucuk et al. (31)	89 (57:36)	Chemoradiotherapy	Non-metastatic	OS; PFS	Yes; Yes	19.7 (Range, 4.0–88.1)
Liang et al. (32)	753 (347:379)	Surgery	Mixed	OS	Yes	NA
Ligorio et al. (33)	57 (29:28)	Taxane/trastuzumab/pertuzumab	Metastatic	OS; PFS	Yes; Yes	36.6
Lin et al. (19)	1,312 (152:1160)	Surgery	Non-metastatic	OS	Yes	78.4 (IQR, 53.1–88)
Mesti et al. (34)	129 (65:64)	Immunotherapy	Metastatic	OS; PFS	No; Yes	22.5
Pérez-Martelo et al. (35)	130 (70:60)	Chemotherapy	metastatic	OS; PFS	Yes; Yes	NA
Provenzano et al. (36)	78 (39:39)	Chemotherapy	metastatic	OS; PFS	Yes; Yes	47.4
Qi et al. (37)	51 (NA:NA)	Neoadjuvant chemoradiotherapy and pembrolizumab	Mixed	PFS	No	20
Sahin et al. (38)	743 (246:351)	Neoadjuvant chemotherapy	Non-metastatic	OS;DFS	No; No	67.5 (Range, 10.5–194.4)
Sato et al. (39)	86 (63:23)	Surgery	Non-metastatic	RFS	Yes	35 (Range, 1–104)
Sato et al. (40)	758 (190:568)	Surgery	Non-metastatic	OS; RFS	Yes; Yes	63.5
Susok et al. (41)	62 (NA:NA)	Immunotherapy	Mixed	PFS	No	NA
Topkan et al. (42)	204 (129:75)	Radiotherapy and temozolomide	Metastatic	OS; PFS	No; No	17.6 (Range, 2:4–108.3)
Topkan et al. (43)	178 (109:69)	Concurrent chemoradiotherapy	Non-metastatic	OS; PFS	Yes; Yes	17.9 (Range, 3.2–104.0)
Wang et al. (44)	89 (34:55)	Surgery	Non-metastatic	DFS	No	29.1 (Range, 4.1–115.8)
Yazgan et al. (45)	114 (57:57)	Androgen receptor-signaling inhibitors	Mixed	OS	Yes	34.6
Yeh et al. (46)	853 (366:487)	Surgery	Mixed	OS;DFS	Yes; Yes	NA
Yekedüz et al. (47)	152 (75:77)	Immunotherapy	Metastatic	OS; PFS	Yes; Yes	29.1
Zeng et al. (48) (training)	53 (27:26)	Immunotherapy and chemotherapy	Mixed	OS; PFS	Yes; Yes	NA
Zeng et al. (48) (validation)	84 (28:56)	Immunotherapy and chemotherapy	Mixed	OS; PFS	Yes; Yes	14

OS, overall survival; DFS, disease-free survival; PFS, progression-free survival; RFS, recurrence-free survival; IQR, interquartile range; NA, not available.

### 3.2. Relationship between the PIV and OS

**Figure 2:**
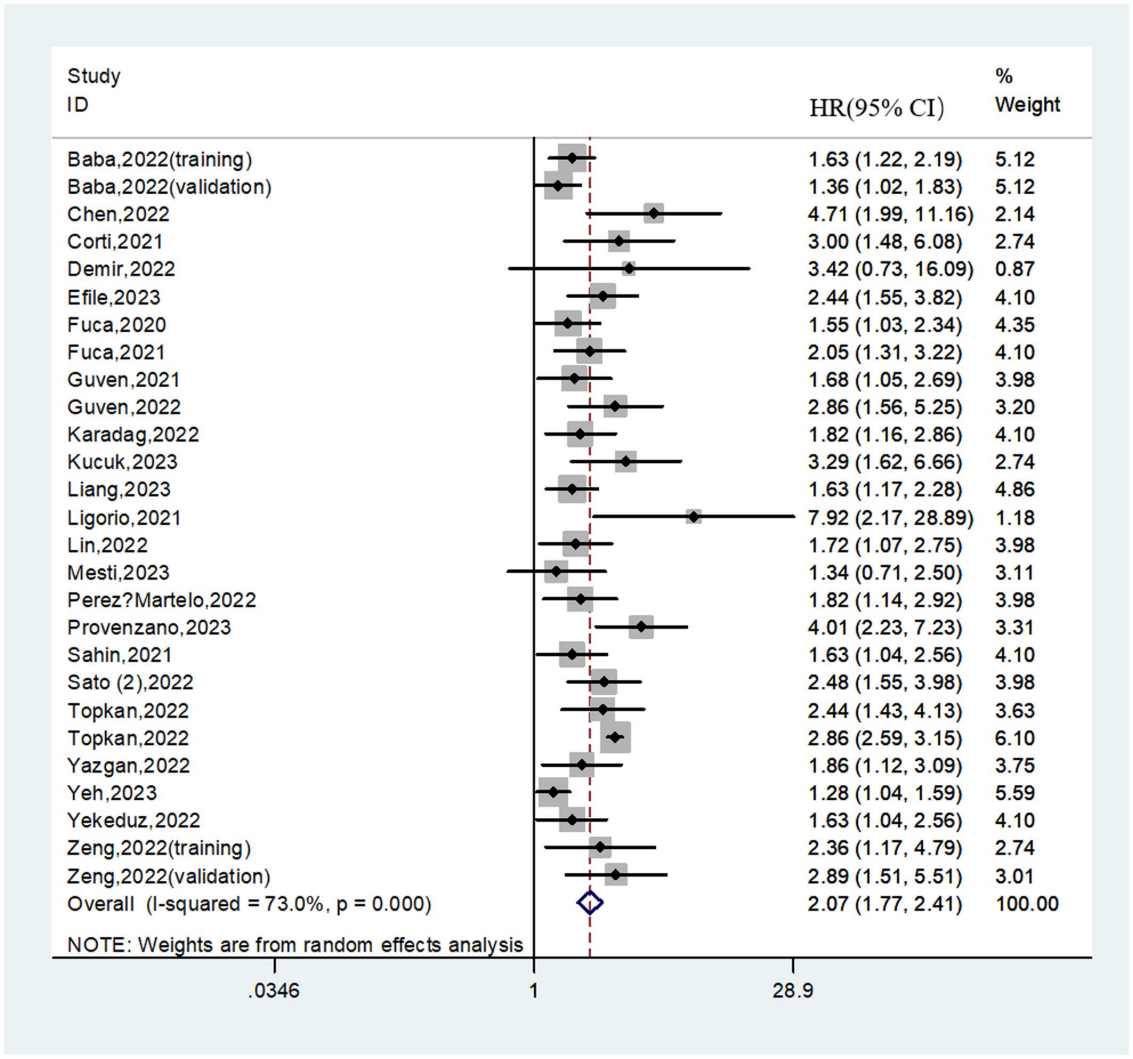
Forest plot assessing the relationship between the PIV and OS.

A total of 8,462 patients from 27 cohorts were included in the pooled analysis of OS. The pooled result revealed that higher PIV predicted poorer OS (HR = 2.07; 95%CI:1.77–2.41; *I*^2^ = 73.0%; Figure 2). Furthermore, subgroup analyses based on country, study center, sample size, cancer type, selection method, cut-off value, treatment strategy, tumor stage, analysis method and follow-up time were performed. As shown in Table 3 and Supplementary Figure S1, the pooled outcomes from all subgroup analyses consistently revealed that patients in the high PIV group had a significantly worse OS compared to those in the low PIV group. In addition, a meta-regression analysis based on these variables was performed to investigate the source of heterogeneity. As shown in Supplementary Table S2, none of these covariates had a significant effect on the hazard ratios of OS (all *p* values>0.05).

**Table 3:** Subgroup analyses for OS of PIV-high patients vs. PIV-low patients.

Subgroup	Cohorts	Patients	Pooled analysis		I square (%)
			**HR**	**95%CI**	
All patients	27	8,462	2.07	1.77–2.41	73.0
Country					
Asian	20	7,239	2.03	1.69–2.43	76.8
Non-Asian	7	1,043	2.23	1.60–3.12	57.5
Study center					
Single center	21	7,483	2.04	1.70–2.44	77.6
Multicenter	6	1,159	2.14	1.63–2.82	26.6
Sample size					
<150	11	1,068	2.34	1.83–3.00	44.8
>150	16	7,574	1.93	1.58–2.36	80.8
Cancer type					
Gastrointestinal	10	3,710	1.96	1.55–2.48	80.3
Breast	5	2,433	2.61	1.56–4.38	63.2
Lung	4	320	3.09	2.15–4.42	0.0
Melanoma	2	357	1.76	1.17–2.63	16.0
Others	6	1,642	1.76	1.37–2.28	51.2
Selection method					
ROC curve	10	4,643	1.97	1.50–2.59	87.5
Median	11	1,396	2.37	1.85–3.03	40.0
MSR	6	2,423	1.81	1.49–2.20	0.0
Cut-off value					
<350	10	5,025	1.75	1.42–2.15	58.6
>350	17	3,437	2.25	1.93–2.62	44.8
Treatment strategy					
Surgery	8	5,089	1.66	1.39–1.98	45.1
Chemo/radiotherapy	8	2059	2.37	1.87–3.01	61.0
Immunotherapy contained	7	929	1.95	1.58–2.39	0.0
Others	4	385	2.71	1.56–4.73	61.7
Tumor stage					
Non-metastatic	**7**	3,583	2.43	1.99–2.96	41.5
Mixed	10	3,180	1.73	1.43–2.09	47.3
Metastatic	10	1,699	2.06	1.64–2.59	43.9
Analysis method					
Univariate	5	1752	1.72	1.40–2.12	0.0
Multivariate	22	6,710	2.14	1.79–2.55	75.9
Follow-up					
<30 months	8	1,076	2.16	1.69–2.76	61.2
>30 months	12	4,617	2.20	1.74–2.77	57.9
NA	7	2,769	1.69	1.39–2.41	38.4

### 3.3. Relationship between the PIV and PFS

**Figure 3:**
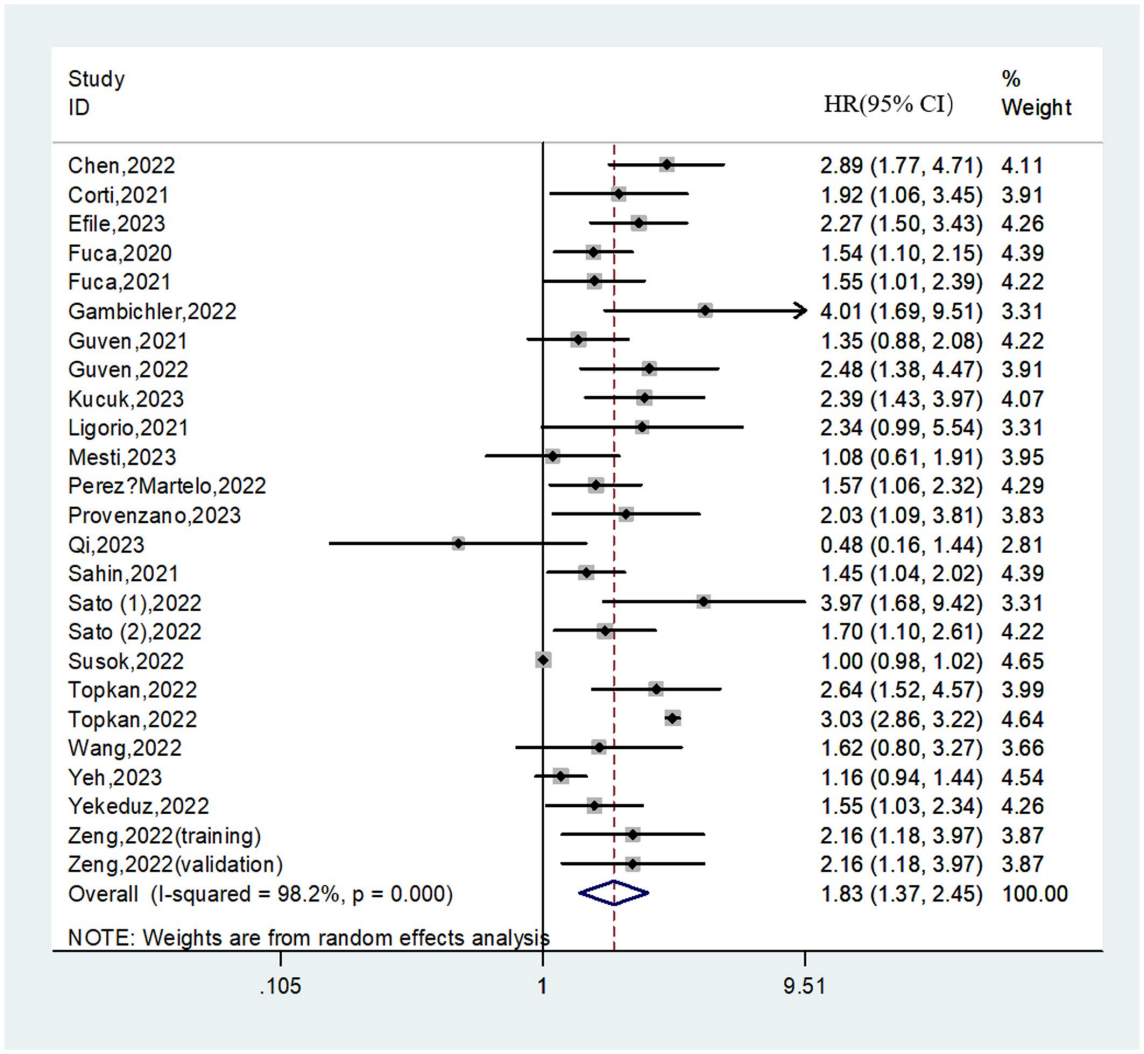
Forest plot accessing the relationship between the PIV and PFS.

In total, 25 cohorts involving 5,391 patients reported on PFS. The pooled HR was 1.83 (95%CI: 1.37–2.45; *I*^2^ = 98.2%), suggesting that higher PIV was associated with a significantly worse PFS (Figure 3). Similarly, subgroup analyses based on above variables were performed due to the significant heterogeneity existed. We found that in almost all subgroups analyses, patients in the high PIV group has an inferior PFS, except for the pooled results from melanoma (HR = 1.13; 95% CI: 0.86–1.47) and univariate analysis (HR = 1.53; 95% CI: 0.99–2.35) (Table 4 and Supplementary Figure S2). Additionally, meta-regression analysis revealed that none of these factors was the source of heterogeneity (all *p* values>0.05; Supplementary Table S2).

**Table 4:** Subgroup analyses for PFS of PIV-high patients vs. PIV-low patients.

Subgroup	**Cohorts**	**Patients**	**Pooled analysis**		**I square (%)**
			**HR**	**95% CI**	
All patients	**25**	5,391	1.83	1.37–2.45	98.2
Country					
Asian	16	4,057	1.93	1.49–2.50	87.8
Non-Asian	9	1,334	1.59	1.20–2.12	79.2
Study center					
Single center	19	4,412	1.81	1.28–2.55	98.6
Multicenter	6	979	1.80	1.48–2.18	0.0
Sample size					
<150	14	1,171	1.80	1.33–2.43	83.7
>150	11	4,220	1.85	1.36–2.50	91.5
Cancer type					
Gastrointestinal	9	2,197	1.89	1.37–2.60	83.3
Breast	3	878	1.63	1.23–2.15	0.0
Lung	4	320	2.43	1.85–3.19	0.0
Melanoma	5	419	1.13	0.86–1.47	50.8
Others	6	1,577	1.81	1.28–2.55	71.3
Selection method					
ROC curve	12	3,361	1.86	1.19–2.91	99.1
Median	8	919	1.93	1.53–2.44	31.9
MSR	5	1,111	1.58	1.32–1.90	0.0
Cut-off value					
<350	7	1957	1.56	1.12–2.16	59.7
>350	18	3,434	1.92	1.35–2.74	98.7
Treatment strategy					
Surgery	5	2090	1.80	1.22–2.64	72.4
Chemo/radiotherapy	8	2059	2.06	1.53–2.78	84.2
Immunotherapy contained	9	1,042	1.40	1.07–1.82	72.3
Others	3	200	2.96	2.02–4.33	0.0
Tumor stage					
Non-metastatic	**9**	2,495	2.29	1.76–3.00	74.0
Mixed	6	1,197	1.46	1.04–2.04	85.2
Metastatic	10	1,699	1.61	1.39–1.87	0.0
Analysis method					
Univariate	6	1,198	1.53	0.99–2.35	83.4
Multivariate	19	4,193	1.91	1.52–2.39	86.5
Follow-up					
<30 months	9	1,096	1.75	1.24–2.49	83.7
>30 months	10	2,844	1.86	1.56–2.22	21.9
NA	6	1,451	1.59	1.16–2.20	86.8

### 3.4. Sensitivity analyses and publication bias

Sensitivity analyses were conducted to assess the robustness of the pooled OS and PFS. After omitting any individual study, pooled HRs with 95% CIs for both OS and DFS were not significantly altered (Supplementary Figure S3).

The Begg’s funnel plots were applied to evaluate the potential publication bias. As shown in Supplementary Figure S4, the funnel plot for PFS was bilaterally symmetric with a Begg’s *p* value of 0.691, indicating that there was no significant publication bias for PFS. While for OS, the Begg’s funnel plot was asymmetric with the *p* value <0.0001, which suggested a high risk of publication bias for this outcome. Trim-and-fill analysis was therefore applied, supplementing a total of 8 unpublished cohorts to balance the funnel plot. Finally, PIV was still associated with inferior OS (HR = 1.82; 95%CI: 1.56–2.13), indicating the robustness of the pooled result.

## 4. Discussion

Cancer-related inflammation is prevalent in patients with malignant diseases, which has been confirmed to promote cancer progression and advancement (6). Traditionally, host inflammation status can be detected through several blood biomarkers, such as neutrophil count, platelet count, and lymphocyte count. Additionally, evidence from numerous studies has demonstrated that their ratios can be applied to predict patient’s short-term and long-term outcomes, especially in cancer patients (7, 8). Importantly, these markers have the natural advantage of being non-invasive, objective, and cost-effective, which provides great potential for their wide clinical applications.

In recent years, a new biomarker, the pan-immune-inflammation value, which consists of serum neutrophil, platelet, monocyte and lymphocyte, has attracted the attention of clinicians due to its promising prognostic significance in several malignancies (12, 30, 39). A recent meta-analysis by Guven et al. (17) has initially demonstrated that high PIV was associated with decreased survival outcomes in cancer patients. Nevertheless, this meta-analysis included only 15 studies (including an abstract) and several common cancer types (such as pancreatic cancer, hepatic cancer and prostate cancer) were not available, which made the prognostic value of PIV in cancer patients still inconclusive. To clarify this issue accurately, an updated meta-analysis including 30 studies with 8,799 cancer patients was performed. Through our quantitatively analyses, we convinced that an elevated PIV markedly predicted poorer OS (HR = 2.07; 95%CI: 1.77–2.41) and PFS (HR = 1.83; 95%CI: 1.37–2.45) in cancer patients. Additionally, benefiting from the inclusion of sufficient studies, we were able to perform detailed subgroup analyses, as well as sensitivity and publication bias analyses It can be seen that the PIV achieved reliable performance in predicting prognosis. Therefore, the PIV may be a valuable and effective inflammatory index to evaluate the oncological outcomes of patients with malignancies.

Dysregulation of inflammatory and immune cells in the tumor microenvironment has been identified as being involved in the tumor progression (49–51). Simultaneously, a higher PIV may result from higher neutrophils, monocytes, and platelets and/or lower lymphocytes. Although the detailed mechanisms of the PIV’s prognostic value in malignancies are unclear, they can be explained as follows: First, neutrophils, as the most common innate immune cells, have been reported to promote tumor invasion and metastasis by secreting VEGFA, MMPs, and other chemokines such as IL-6 and TGF-β (52, 53). At the same time, elevated neutrophils can also cause T cell activation disorders by largely releasing nitric oxide, arginase, and reactive oxygen species, ultimately inhibiting the body’s killing effect on cancer cells (54). Second, monocytes, especially those differentiated into tumor-associated macrophages (TAMs), can induce apoptosis of T cells with antitumor functions (55). In addition, TAM density has been shown to affect tumor tissue angiogenesis by stimulating the production and secretion of pro-angiogenic factors (56, 57). Third, platelets, are reported to induce epithelial–mesenchymal transition and angiogenesis by secreting TGF-β, VEGF and FGF. Moreover, platelets are also able to recruit neutrophils and monocytes, thereby promoting the distant metastasis of tumor cells. Finally, lymphocytes, especially cytotoxic T lymphocytes, play an essential role in cancer immune surveillance and defense (58). It has been reported that high lymphocyte levels in the tumor microenvironment are beneficial for inducing lysis and apoptosis of cancer cells, thereby inhibiting cancer cell proliferation and metastasis (59). On the contrary, lymphopenia has been shown to be associated with a poor prognosis in cancer patients (60).

Notably, the pooled outcomes from subgroup analyses demonstrated that the prognostic value of the PIV for both OS and PFS was consistent in treatment strategies, such as surgery (HR = 1.66 and 1.80), chemo/radiotherapy (HR = 2.37 and 2.06), immunotherapy (HR = 1.95 and 1.40). Given that patients with malignancies would receive one or more anti-tumor treatment strategies, these results showed that PIV could provide prognosis prediction for malignant patients receiving different treatments, especially for those receiving chemo/radiotherapy. In addition, PIV has been shown to have considerable prognostic value across different tumor species, particularly in lung cancer (HR = 3.09 and 2.43). Moreover, the prognostic value of PIV was not affected by the country of publication, cut-off value, and tumor stage, further confirming the clinical universality and efficacy of PIV in cancer patients.

This meta-analysis had several limitations must be acknowledged. First, all of the included studies except one by Qi et al. (37) were designed to be retrospective, which may increase the risk of selection bias. Second, the heterogeneities of pooled outcomes for both OS and PFS were remarkable, even though the subgroup analyses and sensitivity analyses showed consistent results, we failed to find the sources of heterogeneity. Third, significant inconsistencies in the measurement of blood parameters in the included studies, including but not limited to factors such as measurement time, may have contributed to the large variability in the cut-off values of PIV, and may also have had some impact on the confidence of our pooled results. Finally, the cut-off values of PIV varied widely due to various factors such as disease type, population differences, sample size, and detection method, which somewhat limits the clinical use of PIV.

## 5. Conclusion

In conclusion, the present meta-analysis demonstrates an association between elevated pre-treatment PIV and poor survival outcomes in cancer patients. PIV has the potential to be a noninvasive and effective prognostic biomarker for cancer patients.

## Data availability statement

The original contributions presented in the study are included in the article/Supplementary material, further inquiries can be directed to the corresponding author.

## Author contributions

YH-J: Funding acquisition, Conceptualization, Data curation, Formal analysis, Investigation, Methodology, Software, Writing – original draft, Writing – review & editing. RS: Conceptualization, Data curation, Formal analysis, Investigation, Methodology, Software, Writing – review & editing, Visualization. XJ-Q: Funding acquisition, Project administration, Resources, Supervision, Writing – review & editing.

## References

[1] Sung H Ferlay J Siegel RL Laversanne M Soerjomataram I Jemal A . Global Cancer statistics 2020: GLOBOCAN estimates of incidence and mortality worldwide for 36 cancers in 185 countries. CA Cancer J Clin. (2021) 71:209–49. doi: 10.3322/caac.21660, PMID: 33538338

[2] Liu R Zhao K Wang K Zhang L Ma W Qiu Z . Prognostic value of nectin-4 in human cancers: a meta-analysis. Front Oncol. (2023) 13:1081655. doi: 10.3389/fonc.2023.1081655, PMID: 36937394 PMC10020226

[3] Pang HY Chen XF Yan MH Chen LH Chen ZX Zhang SR . Clinical significance of the advanced lung cancer inflammation index in gastrointestinal cancer patients: a systematic review and meta-analysis. Front Oncol. (2023) 13:1021672. doi: 10.3389/fonc.2023.1021672, PMID: 37404758 PMC10316012

[4] Liu H Yang XC Liu DC Tong C Wen W Chen RH. Clinical significance of the controlling nutritional status (CONUT) score in gastric cancer patients: a meta-analysis of 9,764 participants. Front Nutr. (2023) 10:1156006. doi: 10.3389/fnut.2023.1156006, PMID: 37113291 PMC10126262

[5] Diakos CI Charles KA McMillan DC Clarke SJ. Cancer-related inflammation and treatment effectiveness. Lancet Oncol. (2014) 15:e493–503. doi: 10.1016/S1470-2045(14)70263-3 25281468

[6] Singh R Mishra MK Aggarwal H. Inflammation, immunity, and Cancer. Mediat Inflamm. (2017) 2017:1. doi: 10.1155/2017/6027305 PMC5695028 29234189

[7] Diem S Schmid S Krapf M Flatz L Born D Jochum W . Neutrophil-to-lymphocyte ratio (NLR) and platelet-to-lymphocyte ratio (PLR) as prognostic markers in patients with non-small cell lung cancer (NSCLC) treated with nivolumab. Lung Cancer. (2017) 111:176–81. doi: 10.1016/j.lungcan.2017.07.024, PMID: 28838390

[8] Pang Q Zhang LQ Wang RT Bi JB Zhang JY Qu K . Platelet to lymphocyte ratio as a novel prognostic tool for gallbladder carcinoma. World J Gastroenterol. (2015) 21:6675–83. doi: 10.3748/wjg.v21.i21.6675, PMID: 26074706 PMC4458778

[9] Mao S Yu X Shan Y Fan R Wu S Lu C. Albumin-bilirubin (ALBI) and monocyte to lymphocyte ratio (MLR)-based nomogram model to predict tumor recurrence of AFP-negative hepatocellular carcinoma. J Hepat Carcin. (2021) 8:1355–65. doi: 10.2147/JHC.S339707, PMID: 34805014 PMC8594894

[10] Yang XC Liu H Liu DC Tong C Liang XW Chen RH. Prognostic value of pan-immune-inflammation value in colorectal cancer patients: a systematic review and meta-analysis. Front Oncol. (2022) 12:1036890. doi: 10.3389/fonc.2022.1036890, PMID: 36620576 PMC9813847

[11] Karadağ I Karakaya S Yılmaz ME Çakmak Öksüzoğlu ÖB. The potential prognostic novel markers PIV and PILE score to predict survival outcomes at hepatocellular cancer. Eur Rev Med Pharmacol Sci. (2022) 26:7679–86. doi: 10.26355/eurrev_202210_30044, PMID: 36314339

[12] Lin F Zhang LP Xie SY Huang HY Chen XY Jiang TC . Pan-immune-inflammation value: a new prognostic index in operative breast Cancer. Front Oncol. (2022) 12:830138. doi: 10.3389/fonc.2022.830138, PMID: 35494034 PMC9043599

[13] Fucà G Guarini V Antoniotti C Morano F Moretto R Corallo S . The Pan-immune-inflammation value is a new prognostic biomarker in metastatic colorectal cancer: results from a pooled-analysis of the Valentino and TRIBE first-line trials. Br J Cancer. (2020) 123:403–9. doi: 10.1038/s41416-020-0894-7, PMID: 32424148 PMC7403416

[14] Baba Y Nakagawa S Toihata T Harada K Iwatsuki M Hayashi H . Pan-immune-inflammation value and prognosis in patients with esophageal Cancer. Ann Surg Open. (2022) 3:e113. doi: 10.1097/AS9.0000000000000113, PMID: 37600089 PMC10431581

[15] Chen X Hong X Chen G Xue J Huang J Wang F . The Pan-immune-inflammation value predicts the survival of patients with anaplastic lymphoma kinase-positive non-small cell lung cancer treated with first-line ALK inhibitor. Transl Oncol. (2022) 17:101338. doi: 10.1016/j.tranon.2021.101338, PMID: 34999541 PMC8749135

[16] Corti F Lonardi S Intini R Salati M Fenocchio E Belli C . The Pan-immune-inflammation value in microsatellite instability-high metastatic colorectal cancer patients treated with immune checkpoint inhibitors. Eur J Cancer. (2021) 150:155–67. doi: 10.1016/j.ejca.2021.03.043, PMID: 33901794

[17] Guven DC Sahin TK Erul E Kilickap S Gambichler T Aksoy S. The association between the Pan-immune-inflammation value and Cancer prognosis: a systematic review and Meta-analysis. Cancers. (2022) 14:14. doi: 10.3390/cancers14112675 PMC9179577 35681656

[18] Page MJ McKenzie JE Bossuyt PM Boutron I Hoffmann TC Mulrow CD . The PRISMA 2020 statement: an updated guideline for reporting systematic reviews. BMJ. (2021) 372:n71. doi: 10.1136/bmj.n71, PMID: 33782057 PMC8005924

[19] Lin Y Liu Z Qiu Y Zhang J Wu H Liang R . Clinical significance of plasma D-dimer and fibrinogen in digestive cancer: a systematic review and meta-analysis. Eur J Surg Oncol. (2018) 44:1494–503. doi: 10.1016/j.ejso.2018.07.052, PMID: 30100361

[20] Pang HY Yan MH Chen LH Chen XF Chen ZX Zhang SR . Detection of asymptomatic recurrence following curative surgery improves survival in patients with gastric cancer: a systematic review and meta-analysis. Front Oncol. (2022) 12:1011683. doi: 10.3389/fonc.2022.1011683, PMID: 36387075 PMC9643694

[21] Papakonstantinou A Gonzalez NS Pimentel I Suñol A Zamora E Ortiz C . Prognostic value of ctDNA detection in patients with early breast cancer undergoing neoadjuvant therapy: a systematic review and meta-analysis. Cancer Treat Rev. (2022) 104:102362. doi: 10.1016/j.ctrv.2022.102362 35219090

[22] Tierney JF Stewart LA Ghersi D Burdett S Sydes MR. Practical methods for incorporating summary time-to-event data into meta-analysis. Trials. (2007) 8:16. doi: 10.1186/1745-6215-8-16, PMID: 17555582 PMC1920534

[23] Pang HY Liang XW Chen XL Zhou Q Zhao LY Liu K . Assessment of indocyanine green fluorescence lymphography on lymphadenectomy during minimally invasive gastric cancer surgery: a systematic review and meta-analysis. Surg Endosc. (2022) 36:1726–38. doi: 10.1007/s00464-021-08830-2, PMID: 35079880

[24] Higgins JP Thompson SG Deeks JJ Altman DG. Measuring inconsistency in meta-analyses. BMJ. (2003) 327:557–60. doi: 10.1136/bmj.327.7414.557, PMID: 12958120 PMC192859

[25] Demir H Demirci A Eren SK Beypinar I Davarcı SE Baykara M. A new prognostic index in Young breast Cancer patients. J Coll Physicians Surg Pak. (2022) 32:86–91. doi: 10.29271/jcpsp.2022.01.86 34983154

[26] Efil SC Guner G Guven DC Celikten B Celebiyev E Taban H . Prognostic and predictive value of tumor infiltrating lymphocytes in combination with systemic inflammatory markers in colon cancer. Clin Res Hepatol Gastroenterol. (2023) 47:102171. doi: 10.1016/j.clinre.2023.102171, PMID: 37352926

[27] Fucà G Beninato T Bini M Mazzeo L di Guardo L Cimminiello C . The Pan-immune-inflammation value in patients with metastatic melanoma receiving first-line therapy. Target Oncol. (2021) 16:529–36. doi: 10.1007/s11523-021-00819-0, PMID: 34076798

[28] Gambichler T Said S Abu Rached N Scheel CH Susok L Stranzenbach R . Pan-immune-inflammation value independently predicts disease recurrence in patients with Merkel cell carcinoma. J Cancer Res Clin Oncol. (2022) 148:3183–9. doi: 10.1007/s00432-022-03929-y, PMID: 35098389 PMC9508022

[29] Guven DC Erul E Yilmaz F Yasar S Yildirim HC Ercan F . The association between pan-immune-inflammation value and survival in head and neck squamous cell carcinoma. Eur Arch Otorhinolaryngol. (2023) 280:2471–8. doi: 10.1007/s00405-022-07804-x, PMID: 36565325

[30] Guven DC Yildirim HC Bilgin E Aktepe OH Taban H Sahin TK . PILE: a candidate prognostic score in cancer patients treated with immunotherapy. Clin Transl Oncol. (2021) 23:1630–6. doi: 10.1007/s12094-021-02560-6, PMID: 33586122

[31] Kucuk A Topkan E Ozkan EE Ozturk D Pehlivan B Selek U. A high pan-immune-inflammation value before chemoradiotherapy indicates poor outcomes in patients with small-cell lung cancer. Int J Immunopathol Pharmacol. (2023) 37:3946320231187759. doi: 10.1177/03946320231187759, PMID: 37404137 PMC10331221

[32] Liang XL Wei SZ. Predictive value of pan-immune-inflammation value for prognosis of patients with resectable colorectal cancer. Cancer Res Prev Treat. (2023) 50:5. doi: 10.3971/j.issn.1000-8578.2023.23.0150

[33] Ligorio F Fucà G Zattarin E Lobefaro R Zambelli L Leporati R . The Pan-immune-inflammation-value predicts the survival of patients with human epidermal growth factor receptor 2 (HER2)-positive advanced breast Cancer treated with first-line Taxane-Trastuzumab-Pertuzumab. Cancers. (2021) 13:13. doi: 10.3390/cancers13081964 PMC8073809 33921727

[34] Mesti T Grašič Kuhar C Ocvirk J. Biomarkers for outcome in metastatic melanoma in first line treatment with immune checkpoint inhibitors. Biomedicine. (2023) 11:11. doi: 10.3390/biomedicines11030749, PMID: 36979727 PMC10044937

[35] Pérez-Martelo M González-García A Vidal-Ínsua Y Blanco-Freire C Brozos-Vázquez EM Abdulkader-Nallib I . Clinical significance of baseline Pan-immune-inflammation value and its dynamics in metastatic colorectal cancer patients under first-line chemotherapy. Sci Rep. (2022) 12:6893. doi: 10.1038/s41598-022-10884-8, PMID: 35477740 PMC9046216

[36] Provenzano L Lobefaro R Ligorio F Zattarin E Zambelli L Sposetti C . The pan-immune-inflammation value is associated with clinical outcomes in patients with advanced TNBC treated with first-line, platinum-based chemotherapy: an institutional retrospective analysis. Ther Adv Med Oncol. (2023) 15:175883592311659. doi: 10.1177/17588359231165978 PMC10102956 37063779

[37] Qi WX Wang X Li C Li S Li H Xu F . Pretreatment absolute lymphocyte count is an independent predictor for survival outcomes for esophageal squamous cell carcinoma patients treated with neoadjuvant chemoradiotherapy and pembrolizumab: an analysis from a prospective cohort. Thorac Cancer. (2023) 14:1556–66. doi: 10.1111/1759-7714.14898, PMID: 37089116 PMC10260499

[38] Şahin AB Cubukcu E Ocak B Deligonul A Oyucu Orhan S Tolunay S . Low pan-immune-inflammation-value predicts better chemotherapy response and survival in breast cancer patients treated with neoadjuvant chemotherapy. Sci Rep. (2021) 11:14662. doi: 10.1038/s41598-021-94184-7, PMID: 34282214 PMC8289916

[39] Sato R Oikawa M Kakita T Okada T Abe T Tsuchiya H . A decreased preoperative platelet-to-lymphocyte ratio, systemic immune-inflammation index, and pan-immune-inflammation value are associated with the poorer survival of patients with a stent inserted as a bridge to curative surgery for obstructive colorectal cancer. Surg Today. (2023) 53:409–19. doi: 10.1007/s00595-022-02575-8, PMID: 35987967

[40] Sato S Shimizu T Ishizuka M Suda K Shibuya N Hachiya H . The preoperative pan-immune-inflammation value is a novel prognostic predictor for with stage I-III colorectal cancer patients undergoing surgery. Surg Today. (2022) 52:1160–9. doi: 10.1007/s00595-021-02448-6, PMID: 35015151

[41] Susok L Said S Reinert D Mansour R Scheel CH Becker JC . The pan-immune-inflammation value and systemic immune-inflammation index in advanced melanoma patients under immunotherapy. J Cancer Res Clin Oncol. (2022) 148:3103–8. doi: 10.1007/s00432-021-03878-y, PMID: 35006344 PMC9508007

[42] Topkan E Kucuk A Selek U. Pretreatment Pan-immune-inflammation value efficiently predicts survival outcomes in glioblastoma Multiforme patients receiving radiotherapy and Temozolomide. J Immunol Res. (2022) 2022:1–9. doi: 10.1155/2022/1346094 PMC9722312 36479136

[43] Topkan E Selek U Kucuk A Pehlivan B. Low pre-ChemoradiotherapyPan-immune-inflammation value (PIV) measures predict better survival outcomes in locally advanced pancreatic adenocarcinomas. J Inflamm Res. (2022) 15:5413–23. doi: 10.2147/JIR.S385328, PMID: 36158517 PMC9499729

[44] Wang SB Chen JY Xu C Cao WG Cai R Cao L . Evaluation of systemic inflammatory and nutritional indexes in locally advanced gastric cancer treated with adjuvant chemoradiotherapy after D2 dissection. Front Oncol. (2022) 12:1040495. doi: 10.3389/fonc.2022.1040495, PMID: 36387250 PMC9648693

[45] Yazgan SC Yekedüz E Utkan G Ürün Y. Prognostic role of pan-immune-inflammation value in patients with metastatic castration-resistant prostate cancer treated with androgen receptor-signaling inhibitors. Prostate. (2022) 82:1456–61. doi: 10.1002/pros.24419, PMID: 35899494

[46] Yeh CC Kao HK Huang Y Tsai TY Young CK Hung SY . Discovering the clinical and prognostic role of Pan-immune-inflammation values on Oral cavity squamous cell carcinoma. Cancers. (2023) 15:15. doi: 10.3390/cancers15010322 PMC9818418 36612318

[47] Yekedüz E Tural D Ertürk İ Karakaya S Erol C Ercelep Ö . The relationship between pan-immune-inflammation value and survival outcomes in patients with metastatic renal cell carcinoma treated with nivolumab in the second line and beyond: a Turkish oncology group kidney cancer consortium (TKCC) study. J Cancer Res Clin Oncol. (2022) 148:3537–46. doi: 10.1007/s00432-022-04055-5, PMID: 35616728 PMC11800967

[48] Zeng R Liu F Fang C Yang J Luo L Yue P . PIV and PILE score at baseline predict clinical outcome of anti-PD-1/PD-L1 inhibitor combined with chemotherapy in extensive-stage small cell lung Cancer patients. Front Immunol. (2021) 12:724443. doi: 10.3389/fimmu.2021.724443, PMID: 34777341 PMC8586214

[49] Khandia R Munjal A. Interplay between inflammation and cancer. Adv Protein Chem Struct Biol. (2020) 119:199–245. doi: 10.1016/bs.apcsb.2019.09.004 31997769

[50] Mantovani A Allavena P Sica A Balkwill F. Cancer-related inflammation. Nature. (2008) 454:436–44. doi: 10.1038/nature07205 18650914

[51] Tian BW Yang YF Yang CC Yan LJ Ding ZN Liu H . Systemic immune-inflammation index predicts prognosis of cancer immunotherapy: systemic review and meta-analysis. Immunotherapy. (2022) 14:1481–96. doi: 10.2217/imt-2022-0133, PMID: 36537255

[52] Ocana A Nieto-Jiménez C Pandiella A Templeton AJ. Neutrophils in cancer: prognostic role and therapeutic strategies. Mol Cancer. (2017) 16:137. doi: 10.1186/s12943-017-0707-7 28810877 PMC5558711

[53] Xiong S Dong L Cheng L. Neutrophils in cancer carcinogenesis and metastasis. J Hematol Oncol. (2021) 14:173. doi: 10.1186/s13045-021-01187-y, PMID: 34674757 PMC8529570

[54] Jaillon S Ponzetta A di Mitri D Santoni A Bonecchi R Mantovani A. Neutrophil diversity and plasticity in tumour progression and therapy. Nat Rev Cancer. (2020) 20:485–503. doi: 10.1038/s41568-020-0281-y, PMID: 32694624

[55] Mantovani A Schioppa T Porta C Allavena P Sica A. Role of tumor-associated macrophages in tumor progression and invasion. Cancer Metastasis Rev. (2006) 25:315–22. doi: 10.1007/s10555-006-9001-7 16967326

[56] Huang C Li Z Li N Li Y Chang A Zhao T . Interleukin 35 expression correlates with microvessel density in pancreatic ductal adenocarcinoma, recruits monocytes, and promotes growth and angiogenesis of xenograft tumors in mice. Gastroenterology. (2018) 154:675–88. doi: 10.1053/j.gastro.2017.09.039, PMID: 28989066

[57] Ugel S Canè S De Sanctis F Bronte V. Monocytes in the tumor microenvironment. Annu Rev Pathol. (2021) 16:93–122. doi: 10.1146/annurev-pathmechdis-012418-013058 33497262

[58] Gonzalez H Hagerling C Werb Z. Roles of the immune system in cancer: from tumor initiation to metastatic progression. Genes Dev. (2018) 32:1267–84. doi: 10.1101/gad.314617.118, PMID: 30275043 PMC6169832

[59] Pang H Zhang W Liang X Zhang Z Chen X Zhao L . Prognostic score system using preoperative inflammatory, nutritional and tumor markers to predict prognosis for gastric Cancer: a two-center cohort study. Adv Ther. (2021) 38:4917–34. doi: 10.1007/s12325-021-01870-z, PMID: 34379305

[60] Wu ES Oduyebo T Cobb LP Cholakian D Kong X Fader AN . Lymphopenia and its association with survival in patients with locally advanced cervical cancer. Gynecol Oncol. (2016) 140:76–82. doi: 10.1016/j.ygyno.2015.11.013, PMID: 26571200 PMC4782779

