# Supplementary material for: Prognostic significance of the pretreatment pan-immune-inflammation value in cancer patients: an updated meta-analysis of 30 studies
Source: Front Nutr. 2023 Oct 2;10:1259929. doi: 10.3389/fnut.2023.1259929 (PMC10577316; doi:10.3389/fnut.2023.1259929)
Supplement: Supplementary file 1 [file Data_Sheet_1.pdf]

Table S1. Quality assessment of included studies.

Items	Study limitation considered	Long enough follow-up period	Univariate/multivariate analysis used	Predefinition of OS/PFS	Clear cut-off value of PIV	Clear description of including criteria	Clear description of tumor stage/ clinical setting	Patients' consent for research	Clear description of purpose objectives
Baba,2022(training)	1	0	0	1	1	1	1	1	1
Baba,2022(validation)	1	0	1	1	1	1	1	1	1
Chen,2022	1	1	1	1	1	1	1	1	1
Corti,2021	1	0	1	1	1	1	1	1	1
Demir,2022	1	0	0	1	1	1	1	1	1
Efile,2023	1	1	1	1	1	1	1	1	1
Fuca,2020	1	0	1	1	1	1	1	1	1
Fuca,2021	1	0	1	1	1	1	1	1	1
Gambichler,2022	1	0	1	1	1	1	1	1	1
Guven,2021	1	0	1	1	1	1	1	1	1
Guven,2022	1	0	1	1	1	1	1	1	1
Karadag,2022	1	0	1	1	1	1	1	1	1
Kucuk,2023	1	0	1	1	1	1	1	1	1
Liang,2023	1	1	1	1	1	1	1	1	1
Ligorio,2021	1	0	1	1	1	1	1	1	1
Lin,2022	1	1	1	1	1	1	1	1	1
Mesti,2023	1	1	1	1	1	1	1	1	1
Perez-Martelo,2022	1	1	1	1	1	1	1	1	1
Provenzano,2023	1	1	1	1	1	1	1	1	1
Qi,2023	1	1	1	1	1	1	1	1	1
Sahin,2021	1	0	1	1	1	1	1	1	1
Sato (1),2022	1	0	1	1	1	1	1	1	1

Sato (2),2022	1	0	1	1	1	1	1	1	1
Susok,2022	1	1	1	1	1	1	1	1	1
Topkan,2022	1	0	1	1	1	1	1	1	1
Topkan,2022	1	0	1	1	1	1	1	1	1
Wang,2022	1	1	1	1	1	1	1	1	1
Yazgan,2022	1	1	1	1	1	1	1	1	1
Yeh,2023	1	0	1	1	1	1	1	1	1
Yekeduz,2022	1	0	1	1	1	1	1	1	1
Zeng,2022(training)	1	0	1	1	1	1	1	1	1
Zeng,2022(validation)	1	0	1	1	1	1	1	1	1

Table S2. Meta-regression analyses for the association of confounding factors and the hazard ratio for overall survival and progression-free survival.

**Covariates**	Overall survival			Progression-free survival		
	β value	p value	95%CI	β value	p value	95%CI
Country	0.284	0.310	-0.290-0.857	-0.048	0.872	-0.671-0.576
Study center	0.034	0.872	-0.407-0.475	-0.032	0.899	-0.562-0.498
Sample size	-0.097	0.635	-0.520-0.326	0.052	0.810	-0.400-0.503
Cancer type	-0.031	0.534	-0.134-0.072	-0.034	0.667	-0.201-0.133
Selection method	-0.126	0.347	-0.402-0.150	-0.018	0.947	-0.573-0.528
Cut-off value	0.209	0.248	-0.160-0.577	0.121	0.633	-0.409-0.650
Treatment strategy	0.098	0.435	-0.162-0.359	0.125	0.413	-0.193-0.442
Tumor stage	-0.169	0.239	-0.461-0.124	-0.207	0.368	-0.683-0.270
Analysis method	0.086	0.666	-0.328-0.499	0.314	0.266	-0.237-0.896
Follow-up	-0.020	0.862	-0.256-0.217	-0.043	0.758	-0.339-0.252

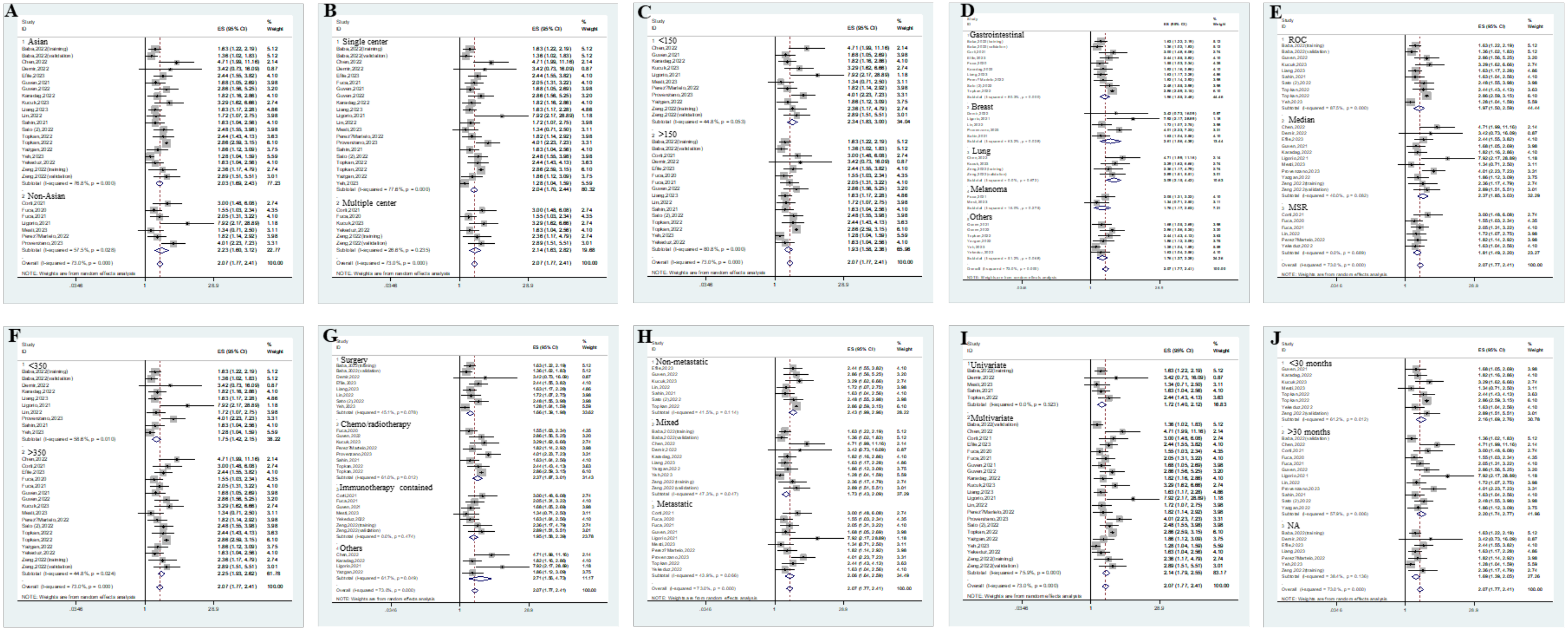

Figure S1. Forest plot of subgroup analyses assessing the relationship between the PIV score and OS. A: Country (Asian vs. Non-Asian); B: Study center (Single center vs. Multiple center) C: Sample size (>150 vs. <150); D: Cancer type (Gastrointestinal vs. Breast vs. Lung vs. Melanoma vs. Others); E: Selection method (ROC vs. Median vs. MSR); F: Cut-off value (<350 vs. >350); G: Treatment strategy (Surgery vs. Chemo/radiotherapy vs. Immunotherapy contained vs. Others); H: Tumor stage (Non-metastatic vs. Mixed vs. Metastatic); I: Analysis method (Univariate vs. Multivariate); J: Follow-up time (<30 months vs. >30 months vs. NA).

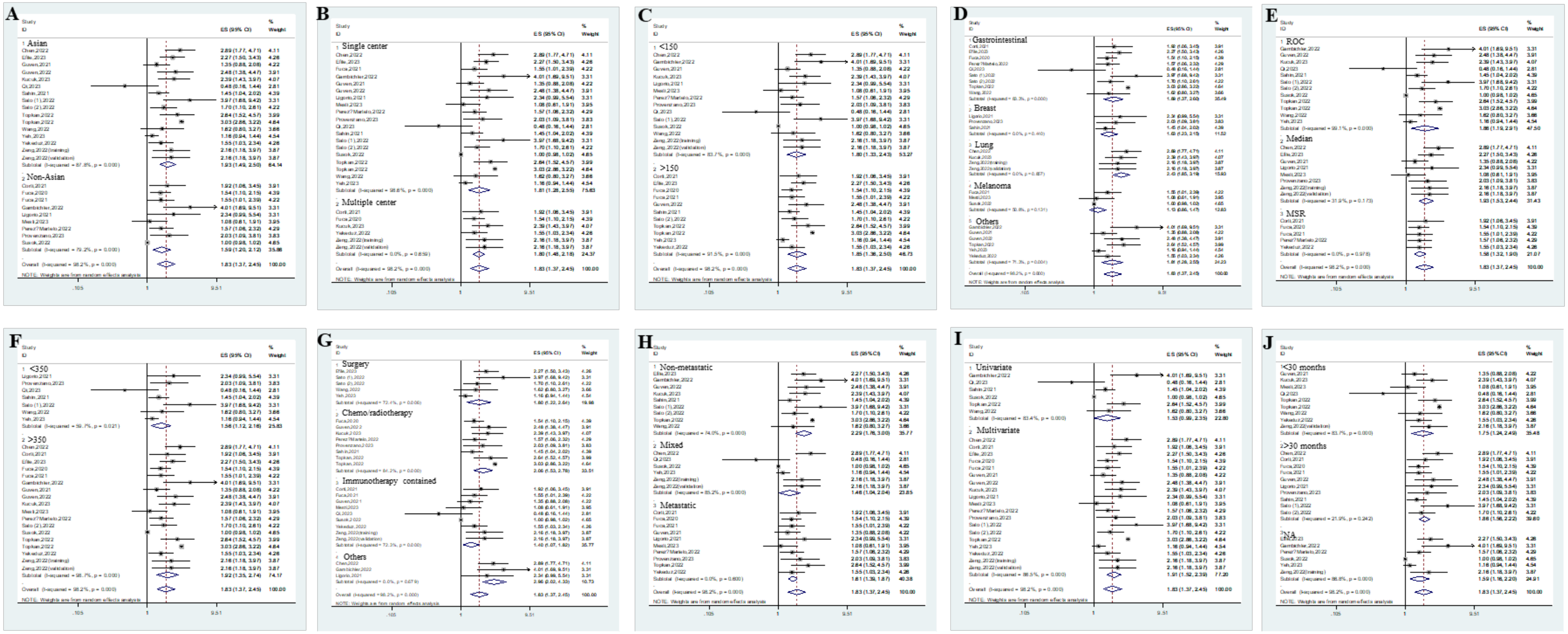

Figure S2. Forest plot of subgroup analyses assessing the relationship between the PIV score and PFS. A: Country (Asian vs. Non-Asian); B: Study center (Single center vs. Multiple center) C: Sample size (>150 vs. <150); D: Cancer type (Gastrointestinal vs. Breast vs. Lung vs. Melanoma vs. Others); E: Selection method (ROC vs. Median vs. MSR); F: Cut-off value (<350 vs. >350); G: Treatment strategy (Surgery vs. Chemo/radiotherapy vs. Immunotherapy contained vs. Others); H: Tumor stage (Non-metastatic vs. Mixed vs. Metastatic); I: Analysis method (Univariate vs. Multivariate); J: Follow-up time (<30 months vs. >30 months vs. NA).

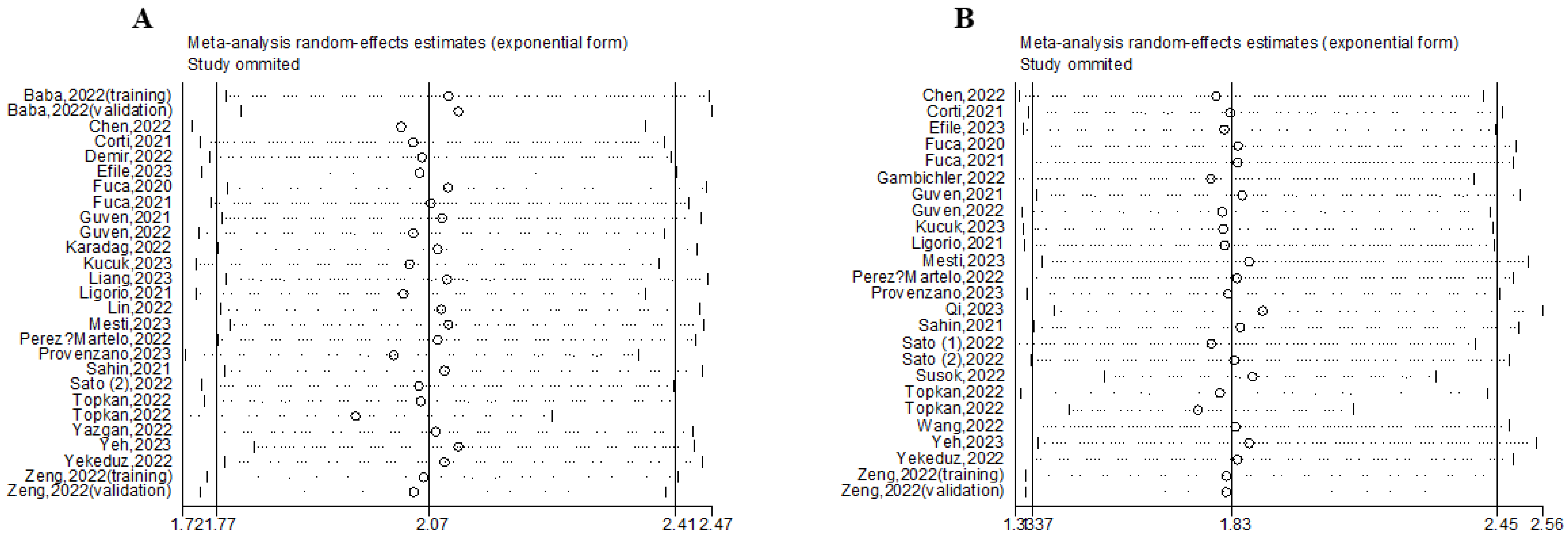

Figure S3. Sensitivity analyses assessing the relationship between the PIV score and survival outcomes including OS (A) and PFS (B).

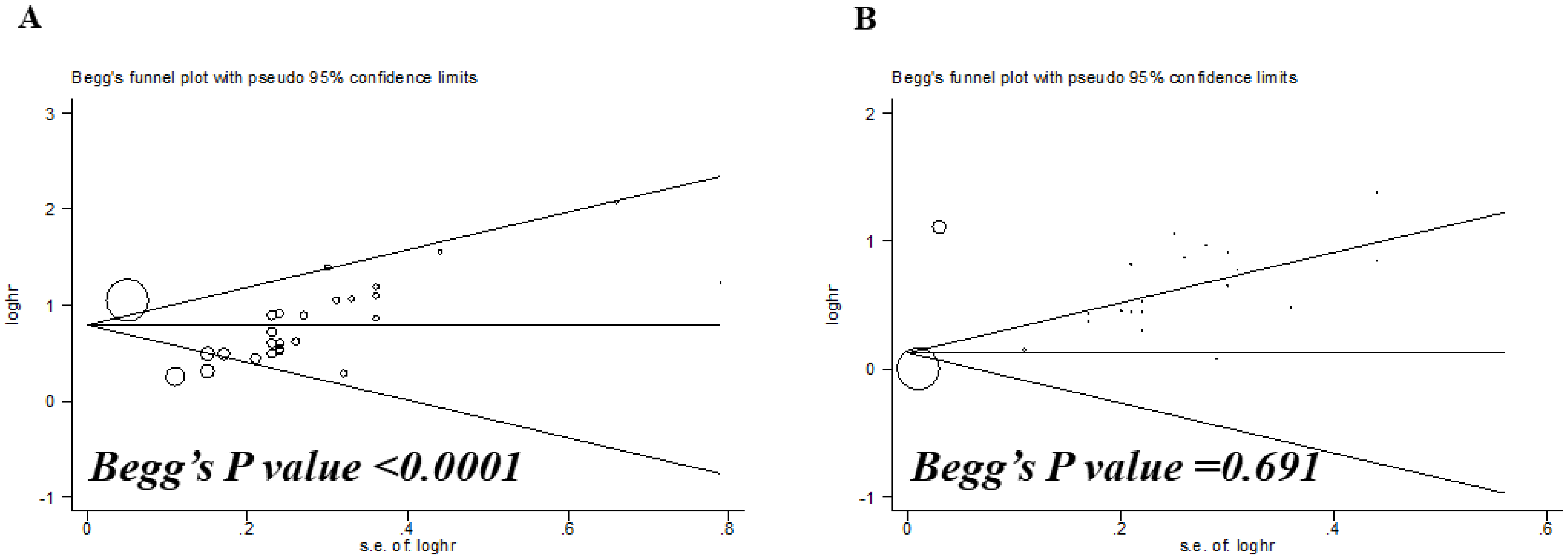

Figure S4. Begg's funnel plot assessing publication bias between PIV and survival outcomes, including OS (A) and PFS (B). The Begg's P values were <0.0001 and 0.691, respectively.
